# Exploring the dynamics of ultrasound training in medical education: current trends, debates, and approaches to didactics and hands-on learning

**DOI:** 10.1186/s12909-024-06092-9

**Published:** 2024-11-14

**Authors:** Florian Recker, Ricarda Neubauer, Yi Dong, Anna Maria Gschmack, Christian Jenssen, Kathleen Möller, Michael Blaivas, Prats Michael Ignacio, Claudia Lucius, Johannes Ruppert, Sophie-Luise Sänger, Roxana Sirli, Johannes Weimer, Susan Campbell Westerway, Constantinos Zervides, Christoph Frank Dietrich

**Affiliations:** 1 Department of Obstetrics and Prenatal Medicine, University Hospital Bonn, Bonn, Germany; 2 University Hospital Bonn, Bonn, Germany; 3 Department of Ultrasound, Xinhua Hospital, Shanghai Jiao Tong University School of Medicine, Shanghai, China; 4 Department for Internal Medicine, Krankenhaus Märkisch Oderland, Strausberg, Germany; 5 Brandenburg Institute for Clinical Ultrasound (BICUS) at Brandenburg Medical University, Neuruppin, Germany; 6 Medical Department I/Gastroenterology, SANA Hospital Lichtenberg, Berlin, Germany; 7 University of South Carolina School of Medicine, Department of Medicine, Columbia, SC USA; 8 The Ohio State University Wexner Medical Center, Department of Emergency Medicine, Columbus, OH USA; 9 Outpatient Department of Gastroenterology, IBD Centre Helios Hospital Berlin Buch, 13125 Berlin, Germany; 10 Department of Medicine, Justus Liebig University Giessen, Giessen, Germany; 11 Medizinische Fakultät Carl Gustav Carus, Technische Universität Dresden, Dresden, Germany; 12 Center for Advanced Research in Gastroenterology and Hepatology, Department of Gastroenterology and Hepatology, ”Victor Babeș” University of Medicine and Pharmacy, Timișoara, Romania; 13 Rudolf Frey Learning Clinic, University Medical Center, Johannes Gutenberg University Mainz, 55131 Mainz, Germany; 14 School of Dentistry & Medical Sciences, Charles Sturt University, Bathurst, Australia; 15 CZMH Medical Physics and Dosimetry Services LTD., Limassol, Cyprus; 16 University Hospital Frankfurt Johann-Wolfgang-Geothe University Frankfurt on the Main Theodor-Stern-Kai, 760596 Frankfurt am Main, Germany

**Keywords:** Ultrasound training, Practical training, Medical education, Hands-on-learning

## Abstract

**Introduction:**

Medical education, especially in ultrasound training, is undergoing significant changes. This study examines practical issues in ultrasound medical education, emphasizing state-of-the-art teaching methods, their effectiveness, and implementation challenges.

**Methods:**

The study analyzed advancements in ultrasound education, including randomized controlled trials comparing peer-to-peer teaching with traditional faculty-led instruction. It evaluated the effectiveness of collaborative small-group training and group size impact in simulation-based training. The study also assessed practical training components outlined in the WFUMB Position Paper Part II.

**Results:**

Findings indicate that peer-to-peer and collaborative small-group training are effective and cost-efficient. Group size did not significantly affect training outcomes. Key practical training elements, including early hands-on sessions and exposure to various equipment, substantially improved learning outcomes. Simulation tools like virtual reality (VR), augmented reality (AR), and phantoms were crucial for teaching standardized procedures and rare pathologies. Challenges identified include developing robust image acquisition skills, scarcity of qualified student tutors, and the need for reliable ultrasound skill assessment methods.

**Conclusion:**

Integrating peer-to-peer teaching and advanced simulation technologies significantly advances ultrasound medical education. These methods enhance cognitive and psychomotor skills but also present challenges such as ensuring quality education and developing effective assessment methods. Future research should compare different simulation tools and establish objective ultrasound proficiency assessment criteria. Continual method evaluation and improvement are essential for effective and standardized ultrasound training, ultimately enhancing patient care and clinical outcomes.

## Introduction

The field of medical education, specifically in the realm of ultrasound training, has undergone significant transformations in recent years. These changes have been driven by the need to provide medical students with comprehensive, practical skills in ultrasound imaging, an increasingly vital tool in diagnostic medicine. This manuscript aims to explore and analyze the various practical issues associated with ultrasound medical education, highlighting the current state-of-the-art teaching methodologies, their efficacy, and the challenges faced in implementing them effectively [1].

Recent advancements in ultrasound education underscore a shift from traditional faculty-led instruction to more dynamic, peer-to-peer teaching methods. This approach has been substantiated by recent randomized controlled trials, demonstrating that the performance of students trained by their peers is comparable to those taught by faculty [2, 3]. A notable study also revealed that a blend of peer-to-peer teaching, combined with blended learning and spaced repetition, surpassed traditional faculty-led methods in basic ultrasound education, particularly in terms of immediate learning outcomes and their sustainability [4].

Moreover, the effectiveness of collaborative training in small groups or pairs, even when compared to single training on simulators, has been highlighted. Such methods are not only more efficient but also less costly and resource-intensive [5, 6]. The underlying success of these collaborative approaches can be attributed to cognitive co-construction of knowledge and the interactive dynamics among trainees.

An interesting aspect of ultrasound training is the size of the groups involved. A recent study indicated that the size of small groups (ranging from 1 to 4 persons) in a short simulation-based obstetric ultrasound course did not significantly affect the outcome [7]. This finding aligns with the recommendation of the World Federation for Ultrasound in Medicine and Biology (WFUMB), which suggests one machine for every 4–5 students in a training session [8].

The practical aspects of ultrasound medical education, as outlined in the WFUMB Position Paper Part II, emphasize several key components [9]. These include early exposure to hands-on sessions, exposure to various equipment and patient positions, training in diverse student groups, continuous assessment opportunities, structured scanning as per guidelines, and access to common pathologies relevant to the students’ skill levels. Furthermore, mastering documentation techniques, understanding regular anatomy, and the ability to perform both fast and slow examinations are considered essential [9]. Eventually, students should progress to performing supervised patient scanning in clinical settings and, post-basic training, access non-supervised training environments such as an ultrasound skills lab.

The role of ultrasound models, often fellow students or tutors, is critical in this educational framework. Direct interaction with peers in a learning context enhances psychomotor and communication skills through immediate verbal feedback. In addition, simulators and phantoms offer standardized teaching and objective comparison, while complex simulation settings (VR, AR, or smartphone-based simulations) allow for the visualization of rare pathologies and emergency scenarios in a safe training environment.

However, challenges remain in this evolving educational landscape. Traditional teaching techniques, such as lectures and seminars, have been found insufficient in developing robust image acquisition skills unless integrated with practical training. The rising demand for ultrasound instruction also highlights a scarcity of qualified student tutors and the need for their continuous education and development. Furthermore, the assessment of ultrasound skills remains a critical area, necessitating reliable methods to ensure the curriculum’s excellence and safeguard patients from inadequately trained clinicians.

In summary, this manuscript delves into the practical aspects of ultrasound medical education, examining the efficacy of current teaching methodologies, their benefits, and the challenges they present. Through this exploration, we aim to provide insights and recommendations for enhancing the quality and effectiveness of ultrasound training in medical education.

## Methods

For this literature review, existing publications in January 2024 on undergraduate ultrasound training were reviewed. The data extraction and synthesis focussed on the developments in practical ultrasound teaching, the development of psychomotor ultrasound skills during hands-on training and the use of simulation tools in student ultrasound education. Results were analysed and discussed in a collaborative interdisciplinary and intergenerational process.

### Practical training and hands-on training

#### Assigned instructors and ultrasound equipment

As direct feedback and supervision of students by an instructor who can verbally and manually guide the student´ probe movements is particularly important in the early stages of learning ultrasound, the numeric ratio of students and lecturers is an important factor. The best feedback would be in a personal 1:1 supervision, which is usually not realistic to implement while aiming to train a high number of students. Striving for a compromise between the optimum learning experience and realistic implementation, the WFUMB recommends a student/teacher-ratio of 1:4 [8]. However, in the view of the fact that many clinics are faced with staff shortages and limited resources available for educational puposes, especially in the form of trained faculty and sufficient ultrasound equipment. For this reason, peer-to-peer learning has become an established approach at many locations. Comparative studies have shown that with pre-existing anatomical knowledge, peer tutors can lead to comparable learning success among students as physician instructors [3, 4, 10, 11]. Furthermore, the investigation of long-term retention of acquired skills also showed no significant difference between students who were taught by peer tutors or by faculty [12, 13]. This opens up the opportunity to relieve the burden on faculty through the use of student peer tutors and at the same time comply with the student-tutor ratio recommended by the WFUMB. However, there are also obstacles associated with peer-to-peer teaching that need to be overcome. Measured against the demand for ultrasound courses, there is also the diffulty of recruiting sufficient peer tutors. Students have to show a high degree of initiative and are often not rewarded financially but work on a voluntary basis as ultrasound tutors. Even with established training programmes, the training of peer tutors requires a lot of practice and time. Training in didactic skills supports the peer tutors, but unfortunately, is not always provided in peer tutor training programmes [3, 10, 14]. Due to the heterogeneity and lack of transparency of peer tutor training programmes, there is often still a lack of confidence in their competence. In addition, they have less clinical expertise and are therefore potentially less able to classify examination findings clinically and make fewer references to everyday clinical practice, for example through personal experiences from clinical cases.

While they are unfortunately usually inferior to lecturers on a professional level due to their age and academic progress, it is precisely this greater cognitive and social proximity to the students that can also be a significant advantage. The temporal proximity to their own starts can help them to better empathise with the students and to choose explanatory approaches that are more accessible to them. In addition, there is often a relaxed atmosphere more quickly, in which students may be more confident to ask questions and less afraid of making mistakes [4, 14, 15]. This significantly encourages more active student participation, which greatly supports learning. In addition to the opportunities that the implementation of peer-to-peer teaching can have on a structural level, it also offers some advantages for the students who decide to become tutors themselves. Through the intensive training during the preparation process, as well as the regular application and explanation of the content during the courses they teach, they usually acquire better ultrasound skills than their fellow students are likely to do on average [14]. In addition, they begin to develop didactic skills - a competence that is not actually taught in medical school but is expected by many in later practice when training residents and students [16]. Nevertheless, the supervision of experienced physicians and didactians and a regular assessment of didactic and practical skills of peer tutors are necessary to ensure high-quality training. As peer tutors usually quit their job with the end of their studies, a medical supervisor in charge of structural and organizational aspects within the skills lab can be useful to ensure continuity beyond changing peer tutor cohorts is needed.

In addition to the limited availability of student ultrasound tutors, limited access to ultrasound equipment is a major problem in practical ultrasound teaching [17]. The number of ultrasound machines often limits the number of students who can participate in ultrasound courses. Less expensive handheld ultrasound devices offer the opportunity to increase the number of devices available for student ultrasound teaching and to allow students more practice time on the device through smaller groups.

### Practice on human models

Ultrasound models who simulate patients are commonly student tutors themselves or students taking the course [18]. Direct interaction with fellow students in the learning context increases the psychomotor and communication skills through immediate verbal feedback [19]. Models with physiological anatomy help learn the basics of the ultrasound device, especially for beginners. When using student models or actors in ultrasound teaching, they should be informed in advance that incidental pathologic findings may occur while they are being scanned for training purposes. In addition, a protocol should be defined in advance, in case this occurs [20]. The student also benefits from the experience of being the model, as they can subsequently better empathize with the patient’s perspective during the examination and have greater sensitivity to their needs or complaints [21]. As fellow students in the same training situation, students are potentially more understanding when mistakes happen during practice, which allows for a more relaxed learning atmosphere, especially for beginners. In addition to practicing on student models, there are also course formats where hands-on practice takes place in the form of clerkships or clinical internships. In this case, students practise on patients under the supervision of a physician [22]. This experience allows the real clinical setting to be practiced and actual pathologies to be seen and discovered. However, it can often be difficult to recruit suitable patients with pathologies for practice courses and there is a lack of reproducibility for exam situations due to individual differences.

### Gamification

In addition to the conventional way of learning practical ultrasound skills by demonstration of the examination by an experienced sonographer followed by the students’ own practice and feedback, gamification can offer a pleasant change from everyday lectures and at the same time effectively promote skills. Elements such as team work, scoring systems, peer competition and prizes are used as incentives to increase the students´ motivation [23]. In a study by Hennekes et al., gamification of ultrasound training led to an increase in students’ self-confidence, perceived autonomy and motivation and promoted their ability to work in a team [24]. On the other hand, gamification can also be particularly competitive, which tends to favour the learning style of previously proactive and motivated students and is even less engaging for more introverted types of learners [24]. Well-known examples of gamification in ultrasound teaching include the Ultrasound Challenge by Bahner et al. [25] or the Sono Slam by Boulger the al. [26].

### Assessment of practical ultrasound skills

Further, choosing an appropriate assessment method is crucial, given the need to closely connect learning objectives, instructional strategies, and exams. Reliable methods for evaluating physicians’ ultrasound skills are critical for training and showing the curriculum’s credibility [27]. To quantify and analyze the success of various attempts to organize ultrasound courses in medical education, the global integration of ultrasonography into medical education will demand uniform assessment and reporting of findings [28, 29]. Assessment has several aims, including optimizing learning and providing immediate feedback to safeguard patients from poorly educated clinicians and re-certifying individuals whose abilities may have deteriorated over time [30, 31]. However, there is no standardized way to assess ultrasound knowledge or examination performance [31–33]. The most used testing modalities in ultrasound training are questionnaires with self-assessments or satisfaction surveys and multiple-choice tests that measure a theoretical understanding of ultrasound abilities. Competency-based modalities such as objective structured clinical exams (OSCE) or directed objective procedural skills (DOPS) are used to explore more practical testing methodologies. Global ratings (e.g., Objective Structured Assessment of Ultrasound Skills (OSAUS) [31]) or even dedicated image evaluation techniques are seldom employed in the existing literature to measure comparable ultrasound talents at a higher level of competence [34].

### Simulation

The proficiency of healthcare professionals in utilizing ultrasound is vital, necessitating robust training methods. Simulation in ultrasound medical education has emerged as a critical tool in this context, providing an effective blend of technology and practical training methodologies. Implementation of ultrasound simulation tools in preclinical and clinical settings could improve the acceptance, practicability and training success for students [8]. According to the World Federation of Ultrasounds in Medicine and Biology (WFUMB) Position Paper on medical student ultrasound education from 2020, the curricular integration of ultrasound education, including the practice on simulators and phantoms in ultrasound skills labs, can provide the urgent needed hands-on experience in ultrasound training [9]. Recent evidence showed that blended learning strategies were an effective adjuct in times of limited access to real-life patients, such as during the beginning of the SARS-CoV-19 pandemic [35].The following detailed examination explores the significance, diverse approaches, and beneficial outcomes associated with the use of simulation in ultrasound training within the medical field.

#### Diverse methods employed in ultrasound simulation training

When talking about simulators, a clear distinction should be made between low-fidelity simulators (for example, custom-made phantoms made of gelatin) and high-fidelity simulators (for example, training mannequins with software) [36]. Virtual and augmentented reality applications represent further technologic advances that also incorporate the clinical situation into the simulation experience. Finally, standardized patients simulating pathologies provide the opportunity to give feedback on interpersonal interactions and examination techniques.

1. **Phantom models**: These tactile models, designed to simulate human tissues, are fundamental in teaching the basics of ultrasound handling and procedures like needle biopsies. They provide a hands-on experience that is crucial for beginners to understand the feel and mechanics of ultrasound probes and their interaction with different tissue types.
2. **High-fidelity simulators**: These sophisticated simulators offer a comprehensive experience by replicating both the physical and physiological aspects of the human body. They are particularly beneficial for training in complex and emergency procedures, allowing learners to encounter rare and critical scenarios in a safe environment.
3. **Virtual reality (VR) and augmented reality (AR) Applications**: The integration of VR and AR in ultrasound training marks a significant leap in simulation technology. These advanced systems offer an immersive learning environment through near-eye visual displays, simulating a range of clinical scenarios with life-like accuracy. Trainees can engage in complex procedures, receive real-time feedback, and experience a variety of pathological conditions in a controlled setting.
4. **Standardized patients with simulated pathologies**: This innovative approach combines the use of actors trained to portray various medical conditions with practical ultrasound practice. It enhances the learning experience by adding a human element, thus improving both technical proficiency and interpersonal communication skills essential in patient care.

#### Benefits and limitations of simulation-based ultrasound training

Empirical research underscores the effectiveness of simulation in enhancing cognitive and psychomotor abilities in ultrasound training. Trainees demonstrate significant improvements in image acquisition, interpretation, and procedural skills. Notably, simulation training fosters confidence and reduces performance anxiety, particularly among novices, facilitating a smoother transition to clinical practice [1]. Moreover, simulation-based training offers a platform for continuous learning and skill assessment. Ultrasound simulation allows students to learn according to their own needs, as for example regarding training speed and repetitions [37]. Receiving feedback on ultrasound skills and the quality of acquired ultrasound images is a crucial part of the progress to develop competence in ultrasound. However, shortages of qualified trainers as well as lack of time reduce the opportunities for individual feedback. In contrast, simulators offer continuous and immediate feedback for the students [38]. Combined mannikin- and computer-based simulation tools are able compare the angle of the acquired images with the angle of a standard image and calculate any differences offering constant feedback on the quality of acquired images. Such techniques enable students to keep track of learning progress and the achievement of learning goals [38]. Supervision and correction by experts can then be applied selectively in later learning stages. This has been shown to be effective in different fields of simulation guided courses in cardiac point-of-care ultrasound (POCUS) and surgical simulation [38, 39].

Mistakes are inevitable parts of any learning process. Therefore simulation-based training should be included into curricula especially for interventional ultrasound procedures [40]. Simulation settings allow learning while making mistakes without patient endangerment. A Danish study on simulation-based obstetric ultrasound training showed that encouraging mistakes during the training compared to the traditional error avoidance strategy resulted in better performance scores and improved transfer into clinical setting [41].

On top of this, simulators also offer the possibility of standardized assessment settings. During training, the learning success can be measured and compared with previous and subsequent training sessions [42]. Final assessments conducted on simulators allow for a high reproducility and objectivity [43]. Ultrasound simulators can be integrated into standardized curricula to assess effectiveness of teaching. In a study by Russell et al., pre-clinical medical students novice to cardiac POCUS were successfully examined on a mannequin with VR ultrasound simulators [44]. Nevertheless, it is important to note that simulators can not completely replace conventional assessment formats to assess actual competence in ultrasound on human beings and are not suitable to evaluaate further criteria as for example the interaction with the patient.

### Simulation of special examinations

Examinations that cross intimate boundaries of the patients or cause any distress or discomfort for the patients, require a high degree of empathy and expertise. However, to spare the patient of uncomfortable situations, there are limited oppurtunities to practice examinations such as gynaecologic and obstretric ultrasound. In these cases, simulator models can serve as a substitute to provide possibilities to learn and apply basic skills.

Simulation-based training is recommended for learning transvaginal ultrasound before examining patients within the clinical setting and results in improved patient satisfaction and shorter examination times [45, 46]. Furthermore, newest high fidelity simulators provide even randomly moving virtual fetus for highly effective ultrasound training in obstetrics [47].

The common challenge of image orientation in transvaginal ultrasound could be taught successfully by simulation sessions in a Danish group of medical students without any ultrasound experience [45]. Likewise, a French multicenter study using simulator training for transvaginal ultrasound for gynecologic emergency showed significantly better scores evaluating image quality in the intervention group [48].

Simulation-based ultrasound training, in addition to clinical ultrasound training, significantly improved obstetrical ultrasound skills in final year students in midwifery, with regard to higher image quality and results after the clinical training [49].

### Simulators in learning anatomy and understanding spatial relationships

Ultrasound simulators can also be used in preclinical anatomy courses. Anatomy training with ultrasound simulators has several advantages compared to traditional formalin-fixed cadavers, as they can be used as long and as often as necessary and provide equal or better understanding of anatomic relationships of surrounding structures [50]. Canty et al. tested the efficiency and feasibility of an ultrasound simulator for self-directed learning of cardiac anatomy compared to cadaver and plastic models in 50 preclinical anatomy students [50]. Only three hours of simulator training appeared equivalent to human cadaver models in mastering multiple-choice-questions and was perceived very positively by the students(50). Both anatomy and ultrasound require complex three-dimensional visual-spatial perception of the students [51]. Understanding real-time two-dimensional ultrasound images and putting them into a three-dimensional context remains a common challenge for ultrasound novices. Ultrasound simulation proved as more useful for orientation within the 3D cardiac anatomy and moreover for understanding the embedment of structures in the surrounding anatomy compared to dissected cadavers. According to a randomized controlled trail by Hu et al. and a systematic review conducted by Halpern et al., using 3D model applications helped students learning ultrasound [52]. VR simulators offer these 3D virtual models in real-time together with the acquired ultrasound images [53].

### Financial considerations

A decisive argument in the question on the acquisition of simulators are their costs. High costs in purchasing and, in some cases, maintenance are to be considered in planning the integration of high fidelity simulators into medical curricula. There is no universal equipment that can serve for all or most of the diverse training landscape. To cover all training needs, especially if the training program covers several different ultrasound indications, a program might have to consider several types of different simulators. Furthermore, the purchase and maintenance of simulation equipment can be costly, and may compete with funds for actual ultrasound equipment or other educational expenses. On the other hand, self-made phantoms, e.g. with gelatin, can replace animal or cadaver models for training ultrasound guided fine needle puncture [54–56]. They can be produced cheaply and effectively [57, 58]. Nevertheless, it should be considered that creating these self-made models can be very time consuming and that those can only be used for a limited number of trainings before they need to be replaced due to the wear and tear of the material caused by the repeated puncturing and the use of materials with a limited shelf life such as gelatin [59]. Studies by Rathbun et al. have investigated gelatin-based compositions and storage options in order to maximize the shelf life of ultrasound phantoms. They found that, stored in the refrigerator, such phantoms can be last for about six weeks before developing microbial growth [60].

### Transferability of simulator-based ultrasound to real humans

Despite the many advantages that simulators have, they cannot replace some aspects of direct human interaction [61]. Most simulation settings fail to mimic the dynamic nature of ultrasound examinations including respiratory movements, pulsation or dynamic tissue properties. The application of examination techniques such as breathing maneuvers to improve image quality or a dynamic examination cannot be imitated by simulators. Therefore, required skills especially for ultrasound interventions are more difficult to transfer from simulation training into practice [62]. Likewise, the additional use of AR technique for training in ultrasound guided biopsies of breast masses did not shorten the time need to perform a successful puncture [63]. However, students considered AR guided ultrasound more relatable with overlaying in situ images onto the anatomy [63]. Furtermore, especially in examination procedures that require a very sensitive handling of the patient’s needs, empathy cannot be practiced. In a study in which transvaginal ultrasound examinations were exercised, students who had previously practiced on real patients outperformed those students who had only trained on simulators [64]. Nevertheless, at least some human attributes could be trained indirectly using VR simulation, with simulated patient histories. Those case vignettes are particularly useful for novice sonographer students prior to clinical practice [65]. However, in the end, neither dissection, cadaver models, virtual models, nor simulation can replace demonstrating dynamic functions in the real clinical setting.

### Psychomotoric skills

Teaching medical ultrasound can be challenging for both the student and tutor, with many factors that must be considered if there is to be a successful outcome. The ultrasound student’s introduction to scanning should be in a supportive and effective learning environment that encourages the student to acquire the complex skill sets needed for clinical practice [66, 67]. If all these elements are present, optimal learning may be achieved (Table 1).

**Table 1:** Tutor/ student relationship – the optimal learning environment

The Tutor	The Student
Wants to teach	Wants to learn
Knows the topic well	Prepared to read about the topic
Has communication skills	Listens to the tutor
Puts the patient first	Asks for assistance if needed
Guides student to advanced scanning skills	Asks questions
Provides feedback	Accepts feedback

To become competent in medical ultrasound requires the student to not only learn the theory behind the modality but also to master image acquisition and interpretation and patient communication. This can only be achieved if the student has access to adequate hands on training to acquire the psychomotor skill sets essential for technical, procedural and clinical competence. Clinical competency can be very subjective and cannot be judged simply by the ability of the student to perform a set sonographic technique but rather experienced assessors need to observe the student scan multiple patients across a range of body shapes and clinical situations.

One of the most prominent groups in the field of ultrasound education has been Nicholls et al., who in 2014 published a landmark paper on psychomotor skills required to perform an ultrasound scan [66]. Learners require both communications, visuospatial and visuomotor skills which can only be obtained through both supervised and independent practice. Many of the scanning skills required are complex, which means they are multi-dimensional and require being broken down into sub-units to enable the learner to better understand the movements required to perform the scanning task to the standard expected for clinical practice. These complex skills can be challenging to both teach and learn and cannot usually be taught in a single teaching session. Obtaining an optimal image of the gallbladder and bile duct is an example of a complex skill as it involves adjusting system depth, focus and gain, colour, transducer pressure and transducer movement, measuring wall and duct thickness as well as communicating with the patient to adjust their position or breathing. There are many gross and fine motor skills required to move the transducer when scanning and these may be difficult for the student to initially understand when watching a scanning demonstration. These movements should be explained by the tutor so as to allow the student to better understand what needs to be practiced.

Using an evidenced based instructional approach is suggested when teaching a complex psychomotor skill. The eleven step skills teaching model, as described by Nicholls et al. [19, 66, 67], ensures that the tutor considers the all aspects related to teaching a complex skill and avoids the risk of cognitive overload for the student when multi dimensional skills are taught in a single teaching session. Breaking down these large skills into sub-sets prior to the teaching session allows for the parts to be taught individually rather than as a whole skill at any one single teaching session. These skill sub-sets are gradually reconstructed until whole task practice is achieved and performed.

The educational steps required to teach the complex psychomotor skills related to ultrasound adapted from Nicholls et al. [66, 67].

1. **Analyse the ultrasound task to be taught**, breaking it down into small itemised teachable steps. Each task should have no more than 7 to 9 sequenced steps. For example teaching how to scan and measure the aorta would require a complete survey scan of the aorta in both sagittal and transverse planes, adjusting system depth, focus and gain, image optimisation, finding the widest point of the aorta in transverse and then accurate caliper placement.
2. **Identify the students ultrasound skill level** and learning needs to enable a focused learning session. If the student is a complete beginner it is important to ensure that they have read relevant learning material related to the skill to be learnt.
3. **Pre-skill conceptualization** where the tutor describes how and when to perform the skill and what the task should look, sound and feel like. This adds to the students pre-reading of learning material.
4. **Demonstration—visualization** is where the tutor silently demonstrates the ultrasound skill with the correct sequence and timing. A silent video clip of the skill may also be used.
5. **Demonstration—verbalization** is where the tutor repeats the ultrasound skill demonstration whilst describing the demonstrated skill steps to the learner.
6. **Verbalization–execution** is where the student describes the ultrasound skill steps with the correct skill sequence and timing in advance of the tutor re-performing the skill. The tutor corrects any incorrectly described skill step as they occur.
7. **Verbalization–performance** is where the student describes the skill steps prior to executing the task steps themselves The tutor withholds feedback.
8. **Limit guidance** and coaching until the completion of the task.
9. **The student performs the ultrasound task** with narration with immediate error correction by the tutor of all the student’s narrated or executed skill errors.
10. **Skill practice** is undertaken in short practice sessions of less than 60 min in duration. Continuing to correct all mistakes during supervised practice until error free performance of the entire task is achieved prevents incorrect techniques becoming a habit.
11. **Post-skill-execution feedback** by the tutor at the completion of the skill session.

### Creating individual learning & skill acquisition plans with students

Scanning skills are developed only through active and experimental learning and the provision of feedback. The core skills required for scanning take time to learn but with dedicated tutors, students would be able to work with their own personal learning plan and be signed off as each core skill is satisfactorily completed [66]. A learning plan (Table 2) assists in guiding the student in acquiring the knowledge, scanning and communication skills required for professional practice. A skill acquisition plan (Table 3) expands on the learning plan to provide a framework for task acquisition and skill stratification. It requires tasks to be broken down into logical steps which will enable the student to progress from simple to complex skills. More importantly it provides task development timeline and goals to accomplish [67].

Learning plans can be simple or complex depending if the learner is a beginner, intermediate or advanced. Any learning plan must be SMART: Specific goal / outcome, Measurable, Achievable, Realistic, Timed interval [68]. The time taken for students to become ‘competent’ in any given area of clinical scanning is highly dependent on how readily the student can complete their learning plan. To develop a learning plan with a student the tutor needs to identify their learning needs, list learning goals and assist the student to develop a plan to meet these goals. They need to discuss what will be learned, how it will be learned, by when, what criteria will be used to evaluate the learning and how will the learning be validated. For example, if teaching how to scan the aorta, we need to ask if the student can correctly use the ultrasound machine with depth, focus, gain, sector, colour and the measuring package. Where do you start & end the aorta scan? Why? What patient position are you going to use? What scan planes are you going to use? How do you measure the aorta?

The student and the tutor need to develop the learning plan together after identifying the learning needs and with fixed achievable goals established. With the student demonstrating good verbal skills that align with the scanning skills, the tutor is more confident that the student is both able to image the correct anatomical structure and identify the same.

The use of student learning and skill acquisition plans for teaching clinical ultrasound has been shown to improve the quality of outcomes for our ultrasound students. By transferring the responsibility for learning to the student, the tutor’s task is made easier, which relates to focused learning and successful students.

**Table 2:** Learning Plan - adapted from Nicholls et al. [67]

Learning Plan	Student Name			Date
Learning goal	Current status	Learning strategies	Required resources	Key performance indicators
What knowledge & skills do I require to achieve this competency?	What level of knowledge & skills do I have now with respect to this learning goal?	How will I reach this learning goal?	What resources do I need to achieve this learning goal?	How can I demonstrate to myself & others that I have achieved this learning goal?
Learner to complete	Learner to complete	Learner to complete	Learner to complete	Learner to complete
By the end of the week I want to be able to correctly scan, image & measure the aorta in long & short axis	I have practiced probe movement & machine controls using an abdominal simulator & observed 10 aorta scans	Review practice guidelines for imaging & measuring the aorta. Describe the scanning & measuring technique to tutor. Scan & measure aorta after tutor has scanned	Access online literature. Tutor scans & measures aorta. Tutor observes student scanning & corrects any mistakes practice aorta scanning & measuring with guidance receive tutor feedback	will be able to image the normal & abnormal aorta & measurements will be the same as tutor pass scanning assessment

**Table 3:** Skill Acquisition Plan - adapted from Nicholls et al. [67]

Skill Acquisition Plan		Student name			Date	
Skill goal	Current skill level	Skill practice strategies	Allocated scan time	Image portfolio	Supervisor comments	Target
to survey scan the aorta in short & long axis	novice	Observe 5 aorta scans. Discuss probe selection & machine presets Describe the scan technique to survey scan the aorta, image a long & short axis & how to measure the aorta Perform a survey scan then image short axis of aorta to measure	10 min	Tutor to observe scanning	Tutor to offer feedback	In the next 2 days, complete 5 scans To include survey scan, long & short axis images & measurement

## Discussion

This review aims to explore the nuances of current teaching methodologies, their effectiveness, limitations, as well as future directions of undergraduate ultrasound education. Recent trends, notably the shift towards peer-to-peer and collaborative learning, have demonstrated significant potential. In comparative studies, peer-to-peer-teaching has proven to be equally effective as faculty-led courses [3, 4, 10, 11]. The assistance provided by student peer tutors can mitigate the limiting effects on the student-tutor ratio and training time caused by staff shortages on the part of ultrasound experts [23]. By increasing social and cognitive congruence, student tutors can also create a more comprehensible way of explaining complex topics that also result in a long-term sustained retention of acquired knowledge [12, 13]. However, the implementation of peer-to-peer teaching approaches also propose challenges. While peers can effectively relay technical skills and practical knowledge, their relative lack of clinical experience may limit the depth of instruction, particularly regaring the clinical relevance of potential findings or practical implications regarding examination techniques. Also, there are certain circumstances, in which the instruction by postgraduate physicians remains superior, e.g. the training of students without any anatomic pre-education or complex ultrasound applications as cardiac or ocular ultrasound [69, 70]. In this regard, ensuring the continuous training of peer tutors in practical ultrasound and didactic strategies can reduce potential differences in training quality and maximise personal and structural benefits. As training peer tutors can have a multiplying effect of fundamental ultrasound skills among students while being less expensive than postgraduate staff, peer-to-peer-concepts can also be an attractive way for clinics in less supplied regions to still offer a cost-effective training program. Furthermore, collaborative learning approaches encourage active participation, critical thinking, and the development of communication skills. Effective interdisciplinary communication is an indispensable clinical skill that can be of crucial importance in clinical work, particularly in stressful situations. A comparison of these two learning methods showed that team-based learning was even more effective in acquiring practical skills in ultrasound, while peer-assisted learning was equivalent in learning theoretical content [71].

Collaborative learning activities, such as case-based or team-based learning as well as simulation scenarios, have the potential to foster teamwork and communication among students from diverse professional backgrounds. Shared simulation sessions allow students to practice performing and interpreting ultrasound scans together, fostering joint decision-making. In disciplines in which the outcome of emergency situations depends crucially on smooth interprofessional communication and cooperation, for example in obstetrics, joint ultrasound courses between medical and midwifery students can facilitate mutual understanding and simultaneously optimize the utilization of resources for overlapping course content [72].

When utilizing peers as ultrasound models in peer-to-peer instruction, sufficient cultural sensitivity is vital, as learners from different backgrounds may have varying attitudes towards learning, communication styles, and physical proximity. Language barriers can impact communication, so clear visuals and possible translation services should be employed. It’s important to respect cultural norms regarding physical contact, especially in practical settings involving ultrasound. Consent and autonomy are critical, as participants should feel comfortable with any physical interactions, and they should have the option to opt out if needed. Especially in the case of incidental findings during training sessions, confidentiality must be maintained.

Simulation technology creates training opportunities where training on humans would be “impractical, dangerous or unethical” [73]. They are a particularly valuable option to learn ultrasound applications that students usually can not practice on each other, such as certain pathologies, gynacologic or obstetric ultrasound. Phantoms, simulators as well as advanced VR/AR technologies offer controlled environment and a safe space where students are allowed to make mistakes. This also allows to practice interventional procedures such as ultrasound-guided punctures or clinical scenarios, including rare and emergency cases. High-fidelity simulators, while offering a more realistic experience, are often more costly. However, simulators alone can be insufficient in achieving sustainable long-term training effects [74]. Nevertheless, the targeted use of innovative simulation technology can be a valuable addition to classic training, especially when it comes to more advanced ultrasound training, interventional ultrasound or the protected simulation of clinical cases to prepare medical students and residents for the complex challegens of modern medicine. Furthermore, by using simulation technology, individual feedback about learning progress can be given based on standardized training and assessment scenarios [42]. Nevertheless, there are several aspects which can´t be sufficiently assessed using a simulator, as for example the use of examination techniques or the direct interaction with the patient. Therefore, simulators can be a beneficical adjunct but can not entirely replace real-life assessments. Implementing advanced ultrasound simulation tools in educational settings with limited resources can also be challenging. In an alternative to high-fidelity simulators, there are a number of methods utilising homemade gelatin models that are appropriate for the practice of fundamental interventional procedures, such as intravenous cannulation [75]. Furthermore 3D-printed anatomical models can also be a cost-efficient alternative [76]. Virtual simulations and open-source software can also be integrated to teach ultrasound techniques in a cost-effective way, offering students case-based learning experiences without requiring physical devices. Institutions may explore partnerships with hospitals or industry for equipment sharing or donations, which can supplement training in resource-limited environments. Mobile and handheld ultrasound devices, which are more affordable than full simulation systems, provide another alternative for practical learning, allowing students to practice essential skills with portable technology.

The Integration of simulation in ultrasound medical education signifies a major leap forward in training methodologies. It addresses the evolving needs for safe, effective, and comprehensive training in the rapidly advancing field of medical ultrasound. On the other hand, the spectrum of fidelity compared to clinical sonography is such that the haptics and ultrasound display of individual simulators are not always representative of scanning on a human. As technological advancements continue, the scope and efficacy of simulation-based ultrasound education is poised to grow, enhancing healthcare delivery standards.

The heterogeneity of current training programs regarding content, extent of courses and subsequent assessment results in wide differences in competences among graduating medical students. On top of this, a high reliance on self-assessment and subjective measures is problematic, potentially leading to overestimation of skills and competencies. While multiple-choice questionnaires (MCQ) enable a high degree of comparability and objectivity through their numerical evaluation, acquired skills in ultrasound should be assessed primarily through practical examination formats. By using standardized examination protocols and as a widely established examination tool, Objective Structured Clinical Examinations (OSCE) are particularly suitable for the examination in undergraduate settings. The embedding in clinical scenarios can reveal further knowledge about indications, pathologies and examination procedures while still maintaining a safe environment for potential mistakes. Standardized assessment protocols and checklists can ensure a high degree of interrater objectivity and comparability. As a workplace-based examination format, Direct Observation of Procedural Skills (DOPS) incorporates further situational skills and the situational fluidity of competence into the assessment, but is often difficult to implement with a higher number of students due to the need for patients.

As faculty-instructed ultrasound courses with healthy models represent the prevailing standard in undergraduate ultrasound education, alternative approaches as for example peer-teaching or simulation-based training concepts are typically evaluated in comparison to the learning outcomes achieved through this instructional method [2, 10]. In most cases, this is accomplished through the administration of theoretical examinations designed to assess the knowledge gained regarding the indications and limitations of ultrasound examinations. Practical assessments, such as OSCEs or DOPS, are an appropriate means of evaluating the actual skills involved in image acquisition and interpretation. While OSCE formats utilize simulated scenarios, DOPS is a workplace-based assessment method involving the evaluation of students in actual clinical settings with real patients [34].

The most common method for evaluating long-term skill retention is to conduct a follow-up assessment at the designated interval following the initial training assessment. For good comparability, the utilized assessment method then often is the same as chosen before [77].

Looking forward, it is essential to address these challenges through continued research and innovation in teaching methodologies. A systematic investigation and completion of the evidence with regard to the comparative evaluation of didactic methods and their long-term effects as well as the subsequent analysis of the study results in the form of meta-analyses form the basis for the formulation of guidelines for undergraduate courses, as international ultrasound societies, the EFSUMB and WFUMB already published position papers [6, 8, 9]. These can serve as a concrete template for universities and training centers when planning and implementing ultrasound courses. Furthermore, a stronger representation of ultrasound in the requirements of national competence catalogs can promote the uniform implementation of ultrasound teaching at universities. With regard to the most effective planning of an already overloaded curriculum, methods that promote the highest possible retention of acquired skills in the long term are particularly useful. In future, these questions should be given greater consideration in the design of ultrasound teaching studies.

## Conclusion

The integration of peer-to-peer teaching and advanced simulation technologies in ultrasound medical education represents a significant advancement in training methodologies. This approach not only addresses the practical issues of safety, standardization, and resource utilization but also fosters an interactive and collaborative learning environment. The evidence supporting the efficacy of these methods in enhancing cognitive and psychomotor skills is promising.

However, this evolution in teaching strategies brings forth new challenges. The need for well-trained peer tutors, standardization in course design, and effective assessment methods are crucial areas that require attention. Future research should focus on comparing the effectiveness of different simulation tools and developing objective assessment criteria to measure the proficiency of ultrasound skills.

In summary, while the current trends in ultrasound medical education are encouraging, there is a clear need for continuous evaluation and improvement of these methods. By addressing the challenges and capitalizing on the benefits of modern teaching strategies, the field can move towards a more effective and standardized approach to ultrasound training, ultimately enhancing patient care and clinical outcomes.

## Data Availability

Data available within the article or its supplementary materials.
